# Supplementary material for: B Cells in Breast Cancer Pathology
Source: Cancers (Basel). 2023 Feb 28;15(5):1517. doi: 10.3390/cancers15051517 (PMC10000926; doi:10.3390/cancers15051517)
Supplement: Supplementary file 1 [file cancers-15-01517-s001.zip › cancers-2146777-supplementary.pdf]

Supplementary Table S1. Cohorts collected for BLESS

PMID	Year	Patients	Tissue	Reference
29961579	2018	8 breast cancer patients (including ER+, HER2+, PR+, TNBC)	Primary tumour, LNs, normal tissue, blood	Azizi *et al.* [1]
32790115	2020	5 TNBC patients	Primary tumour	Wu *et al.* [2]
32949350	2020	2 breast cancer patients from nab-paclitaxel and pembrolizumab treatment (1 responder and 1 progressor)	Tumour core biopsy	Deng *et al.* [3]
33727227	2020	13 samples from 7 patients (4 BRCA1 mutation carriers and 3 non carriers)	Adjacent normal mammary from the ipsilateral breast and contralateral normal mammary	Hu *et al.* [4]
33950524	2021	28 normal or preneoplastic BRCA+/- and 34 tumours with ER+, HER2+ and TNBC including male tumours	Normal tissue, tumours and LNs	Pal *et al.* [5]
34493872	2021	11 ER+, 5 HER2+, 10 TNBC	Primary tumour	Wu *et al.* [6]
34611125	2021	1 Luminal B, 2 HER2+, 2 TNBC patients	Primary tumour, LNs	Xu *et al.* [7]
34653365	2021	22 patients with advanced TNBC treated with paclitaxel or its combination with the anti-PD-L1 atezolizumab	Biopsy samples for primary tumour, LNs, liver or lung	Zhang *et al.* [8]
33564857	2021	1 luminal B breast cancer patient	Primary tumour, negative LNs, positive LNs	Xu *et al.* [9]
36129636	2022	1 TNBC	Primary tumour, LN	Liao *et al.* [10]
34992092	2022	5 breast cancer patients	Primary tumour and paired sentinel LNs	Jiao *et al.* [11]
36040519	2022	8 breast cancer patients	Primary tumour, adjacent normal tissue, LNs and PBMC	Mao *et al.* [12]
36357424	2022	8 patients (5 luminal subtypes and 3 HER2-overexpressing)	Primary tumour and paired LN metastasis	Liu *et al.* [13]

ER: estrogen receptor; HER2: human epidermal growth factor receptor 2; TNBC: triple negative breast cancer; LN: lymph node; PBMC: peripheral blood mononuclear cell.

References

1. Azizi, E., et al., *Single-Cell Map of Diverse Immune Phenotypes in the Breast Tumor Microenvironment.* Cell, 2018. **174**(5): p. 1293-1308 e36.
2. Wu, S.Z., et al., *Stromal cell diversity associated with immune evasion in human triple-negative breast cancer.* EMBO J, 2020. **39**(19): p. e104063.
3. Deng, J., et al., *Serial single-cell profiling analysis of metastatic TNBC during Nab-paclitaxel and pembrolizumab treatment.* Breast Cancer Res Treat, 2021. **185**(1): p. 85-94.
4. Hu, L., et al., *Single-Cell RNA Sequencing Reveals the Cellular Origin and Evolution of Breast Cancer in BRCA1 Mutation Carriers.* Cancer Res, 2021. **81**(10): p. 2600-2611.
5. Pal, B., et al., *A single-cell RNA expression atlas of normal, preneoplastic and tumorigenic states in the human breast.* EMBO J, 2021. **40**(11): p. e107333.
6. Wu, S.Z., et al., *A single-cell and spatially resolved atlas of human breast cancers.* Nat Genet, 2021. **53**(9): p. 1334-1347.
7. Xu, K., et al., *Single-cell RNA sequencing reveals cell heterogeneity and transcriptome profile of breast cancer lymph node metastasis.* Oncogenesis, 2021. **10**(10): p. 66.
8. Zhang, Y., et al., *Single-cell analyses reveal key immune cell subsets associated with response to PD-L1 blockade in triple-negative breast cancer.* Cancer Cell, 2021. **39**(12): p. 1578-1593 e8.
9. Xu, K., et al., *Integrative analyses of scRNA-seq and scATAC-seq reveal CXCL14 as a key regulator of lymph node metastasis in breast cancer.* Hum Mol Genet, 2021. **30**(5): p. 370-380.
10. Liao, N., et al., *Single-cell profile of tumor and immune cells in primary breast cancer, sentinel lymph node, and metastatic lymph node.* Breast Cancer, 2022.
11. Jiao, S., et al., *Intratumor expanded T cell clones can be non-sentinel lymph node derived in breast cancer revealed by single-cell immune profiling.* J Immunother Cancer, 2022. **10**(1): p. e003325.
12. Mao, X., et al., *Single-cell and spatial transcriptome analyses revealed cell heterogeneity and immune environment alternations in metastatic axillary lymph nodes in breast cancer.* Cancer Immunol Immunother, 2022.
13. Liu, T., et al., *Single cell profiling of primary and paired metastatic lymph node tumors in breast cancer patients.* Nat Commun, 2022. **13**(1): p. 6823.
